# Phosphodiesterase 4 Inhibitors in Allergic Rhinitis/Rhinosinusitis

**DOI:** 10.3389/fphar.2020.01135

**Published:** 2020-07-22

**Authors:** Viera Janosova, Vladimir Calkovsky, Heiko Pedan, Estera Behanova, Andrej Hajtman, Andrea Calkovska

**Affiliations:** ^1^ Clinic of Otorhinolaryngology and Head and Neck Surgery, Jessenius Faculty of Medicine, Comenius University in Bratislava, and Martin University Hospital, Martin, Slovakia; ^2^ Department of Physiology, Jessenius Faculty of Medicine, Comenius University in Bratislava, Martin, Slovakia

**Keywords:** allergic rhinitis, PDE4 inhibitor, inflammation, T helper cells, immunomodulation

## Abstract

Allergic rhinitis/rhinosinusitis (AR) is the most common allergic disease. It affects patients’ quality of life and may influence the severity of lower airway disease such as asthma. Therefore, its treatment is of great importance. AR is treated by a combination of effective approaches; however, in some patients, the disease is uncontrolled. In the last several years, the concept of AR has shifted from increased T helper 2 (Th2) cell signaling and downstream inflammation to disease phenotypes with non-Th2-mediated inflammation. AR is a largely heterogenous group of airway diseases, and as such, research should not only focus on immunosuppressive agents (e.g., corticosteroids) but should also include targeted immunomodulatory pathways. Here, we provide an overview of novel therapies, focusing on the role of phosphodiesterase-4 (PDE4) inhibitors in AR. PDE4 inhibitors are potent anti-inflammatory agents that are used for the treatment of inflammatory airway diseases including AR. The PDE4 inhibitor roflumilast was shown to effectively control symptoms of AR in a randomized, placebo-controlled, double-blinded, crossover study in patients with a history of AR. However, only a few PDE4 inhibitors have proceeded to phase II and III clinical trials, due to insufficient clinical efficacy and adverse effects. Research is ongoing to develop more effective compounds with fewer side effects that target specific inflammatory pathways in disease pathogenesis and can provide more consistent benefit to patients with upper airway allergic diseases. Novel specific PDE4 inhibitors seem to fulfill these criteria.

## Introduction

Allergic rhinitis (AR) is an inflammatory disorder of the nasal epithelium. Anterior and posterior rhinorrhea, nasal congestion, sneezing, and itching are typical clinical symptoms, which are reversible spontaneously or in response to therapy (Graft, 2000).

AR is not considered to be the most serious medical condition compared with other respiratory diseases. However, patients with AR have a reduced quality of life, lack of or worsened quality of sleep, cognitive function impairment, irritability, and fatigue, especially during peak pollen season (Brożek et al., 2017). The most common form of AR is caused by exposure of sensitized patients to allergens such as house dust mites, grass pollen, tree pollen, weed pollens, cat and dog fur, and mold (Graft, 2000).

The phenotypes of this disease appear to actively change, with about 50–70% of patients suffering from mixed rhinitis (non-AR and AR) and approximately 10–20% suffering from the pharmacologically resistant phenotype. AR with localized nasal allergic response in the absence of systemic atopy has also been identified (Price et al., 2015).

Standard treatment of AR includes the combination of oral antihistamines and glucocorticoids (GCs), which may not provide sufficient relief from symptoms; thus, novel therapies are needed (Tabatabaian and Casale, 2018). In recent years, various AR phenotypes caused by non-T helper 2 (Th2) cell-mediated inflammation have been identified. Therefore, it is important to develop more effective drugs with different mechanisms of action such as inhibition of Janus kinase, phosphodiesterase (PDE), and chemoattractant receptor homologous molecule of Th2 cells (Howell et al., 2018).

PDEs are responsible for the hydrolysis of intracellular cyclic adenosine monophosphate (cAMP) and cyclic guanosine monophosphate (cGMP), which have emerged as novel therapeutic targets. PDE inhibitors allow the elevation of cAMP and cGMP, which leads to a variety of cellular effects such as relaxation of airway smooth muscle and inhibition of cellular inflammation. Special attention has been paid to PDE4 inhibitors, which are potent anti-inflammatory agents that have shown encouraging results in the treatment of chronic obstructive pulmonary disease (COPD) and asthma. Their potential in the management of upper airway diseases has also been suggested. This review summarizes current evidence on the beneficial as well as harmful effects of the clinical use of PDE4 inhibitors in allergic rhinitis/rhinosinusitis (Contreras et al., 2017).

## Definition and Epidemiology of AR

AR is clinically defined as a symptomatic disorder of the nose induced after allergen exposure by immunoglobulin E (IgE)-mediated inflammation of the nasal membranes (Fokkens et al., 2020). AR is an extremely common disease that persists throughout life (Brożek et al., 2017) and occurs very frequently. Real-time data are difficult to interpret. Statistics only show physician-diagnosed AR, but the number of patients suffering from AR is a bit higher (Skoner, 2001). During the last 20–30 years, the prevalence of allergic diseases has increased (Howell et al., 2018). The prevalence of self-reported AR has also increased and is approximately 2%–25% in children and 1% to more than 40% in adults. The prevalence of confirmed AR in adults oscillates in Europe from 17% to 28.5% (Brożek et al., 2017), meaning that 100–150 million individuals in Europe suffer from AR (Price et al., 2015). In the United States, 10–30% of the population has been diagnosed with AR (Cingi and Muluk, 2020). In Asia, AR affects a large part of the population, from 27% in South Korea to 32% in the United Arab Emirates (Chong and Chew, 2018). The incidence of AR seems to be comparable worldwide. AR is frequently associated with asthma; 15–38% of patients with AR have asthma and nasal symptoms are present in 6–85% of asthmatic patients. Thus, AR is a risk factor for asthma and successful management of AR can improve asthma symptoms (Brożek et al., 2017).

## Pathophysiology of AR

As aforementioned, the most causal antigens for AR are inhalant allergens such as house dust mites, animal dander, and pollen. Many allergens have protease activity and are able to impair the epithelial barrier and penetrate into nasal mucosa. Allergen-specific Th2 cells are produced in patients with AR, whereas allergen-specific Th1 cells are produced in healthy individuals (Okano, 2009). The results of long-term studies have shown that all allergic diseases are primarily characterized by enhanced Th2 pathway activation, increased serum levels of IgE, blood eosinophil count, and allergen reactivity (Zheng et al., 2011). However, in the last several years, the concept of AR has shifted from increased Th2 signaling and downstream inflammation to disease phenotypes with non-Th2 mediated inflammation. AR is a largely heterogenous group of airway diseases, and as such, research should not only focus on immunosuppressive agents (e.g., corticosteroids) but should also include targeted immunomodulatory pathways (Okano, 2009).

Basophil cells produce cytokines such as interleukin 4 (IL-4) and thymic stromal lymphopoietin in response to allergens with protease activity, and may contribute to Th2 differentiation (Sokol et al., 2008). Th2 cells produce IL-4/IL-13 and express CD40 ligand, which are responsible for converting B cells to IgE-producing B cells (Okano, 2009). Inhalation of allergens by sensitized patients is followed by antigen passing through the epithelial tight junctions in the nasal mucosa and binding to IgE receptors on the surface of mast cells in the nasal mucosa. Chemical mediators such as histamine and prostaglandins are released. Histamine increases paracellular permeability by regulating tight junctions through coupling with H1 receptors (Okano, 2009). Interactions among chemical mediators, sensory nerve terminals, and blood vessels are responsible for the early phase of AR (sneezing, rhinorrhea, nasal congestion) (Gelfand, 2004). Late-phase inflammation develops within 6–10 h after allergen stimulation (Okano, 2009).

Dysregulation of dendritic cells (DCs) plays an important role in the pathogenesis of AR. Understanding DC biology and its functions in physiologic and pathologic conditions may provide an understanding of the role of DCs in the initiation and perpetuation of inflammatory sinonasal diseases, and also lead to the development of potentially novel therapeutic strategies targeting DCs (Cao et al., 2016). DCs influence inflammation and adaptive responses of the body by releasing pro-inflammatory cytokines and other mediators. They are antigen-presenting cells (APCs) able to polarize T effector cells.

Activation of the cytotoxic activity of natural killer and CD8^+^ T cells protects against intracellular pathogens, but can also lead to tissue injury. DCs produce IL-23, and together with IL-6 and IL-1β, are involved in the expansion of Th17 cells, which are responsible for strong pro-inflammatory responses against extracellular pathogens, possibly leading to chronic inflammation and autoimmune diseases (Gianello et al., 2019). Thus, for novel therapeutic strategies, it is important to focus on pathological conditions characterized by the expansion of Th1 and/or Th17 responses (Gianello et al., 2019).

## Therapy

The primary non-pharmacological strategy in AR management is avoiding allergens and irritants (Ridolo et al., 2016). In relation to the elimination of allergens, topical saline irrigations help to reduce symptoms of AR when used alone or together with other drugs (Ghadersohi and Tan, 2017).

As first-line therapy for AR, GCs and antihistamines administered orally or intranasally are used. GCs have become a clinical mainstay for the suppression of numerous inflammatory and autoimmune diseases. They reduce the infiltration of inflammatory cells into the nasal mucosa, maturation of immunocompetent cells, cytokine production, and mediator release by mast cells (Hox et al., 2020). They also inhibit histamine release, induce apoptosis of eosinophils, and reduce the recruitment of APCs (Okano, 2009). Inhibition of B cells through GCs is limited, but GCs are able to inhibit class-switching to IgE in the nasal mucosa (Till et al., 2001). GCs are also known as anti-inflammatory drugs that affect nasal constitutive, epithelial, vascular endothelial cells, and glands (Okano, 2009). GCs signal through genomic and non-genomic pathways (Ramamoorthy and Cidlowski, 2016). The classic, genomic actions of GCs are mediated through a single GC receptor (GR) with many isoforms, which expands the heterogeneity of hormonal signaling. GRs are expressed throughout the body and are localized in the cytoplasm of target cells. The main anti-inflammatory effects of GC are based on their ability to inhibit the transcription of cytokine and chemokine genes participating in inflammation (Parnham et al., 2019).

Three generations of antihistamines that specifically block the effects of histamine on histaminergic receptors are available. First-generation oral antihistamines such as cyproheptadine are no longer recommended, because of their sedative and anticholinergic effects. Second-generation antihistamines such as loratadine or rupatadine lead to good improvements in symptoms without side effects to the central nervous system. They are rapid and well tolerated for the symptomatic treatment of AR (Graft, 2000).

The combination of oral antihistamines and intranasal corticosteroids is a reasonable choice because of the significantly faster onset of treatment effects. However, the combination of intranasal antihistamines and intranasal corticosteroids does not alleviate symptoms as well as the combination of oral antihistamines with intranasal corticosteroids (Brożek et al., 2017).

During the above-mentioned long-term therapy, some side effects can occur such as atrophy of the nasal mucosa or systemic side effects, especially in children (Dahl, 2006). Antihistamines can cause drowsiness or prolongation of the QT interval on electrocardiogram (Schmidt et al., 2001). Common pharmacological treatment with intranasal steroids, oral or intranasal antihistamines does not completely relieve the symptoms of AR (Tabatabaian and Casale, 2018). This is why more specific substances for disease control are needed.

## PDES as a Therapeutic Target

PDEs are a group of enzymes consisting at least of 11 members that cause hydrolysis and subsequent inactivation of cAMP and cGMP serving as intracellular second messengers (Howell et al., 2018; Mokry et al., 2018). They modulate many cellular functions such as contractility of the myocardium, airway and vascular smooth muscle tone, platelet aggregation, and the release of inflammatory mediators (Soto and Hanania, 2005). PDE3, PDE4, PDE5, and PDE7 isoforms are the most well studied selective PDEs in relation to immune responses in the respiratory system (Mokry and Mokra, 2013).

PDE4 is the major cAMP-metabolizing enzyme expressed inside the inflammatory cells. The PDE4 family in mammals consists of four subfamilies, encoded by four genes (*PDE4A, PDE4B, PDE4C*, and *PDE4D*). *PDE4A*, *PDE4B*, and *PDE4D* are expressed by neutrophils, eosinophils, B cells, T cells, DCs, monocytes, and macrophages. Expression of *PDE4C* is typically minimal or absent (Contreras et al., 2017). Expression is triggered by epithelial damage, microbial invasion, and allergen sensitization. Its inhibition is a promising target for suppressing inflammatory responses (Contreras et al., 2017) as was demonstrated for roflumilast in an animal model of acute lung injury (Kosutova et al., 2018). PDE4 inhibitors are well-characterized pharmaceutical agents with a broad range of anti-inflammatory activities also in various inflammatory conditions including allergic diseases (Howell et al., 2018).

As aforementioned, PDE4 inhibitors may serve as potential therapeutic agents for various respiratory diseases, as well as for non-Th2 mediated AR. These effects have been evaluated *in vitro* and in animal models of allergic asthma. Their anti-inflammatory activity results from blocking the degradation of cAMP in lymphocytes, eosinophils, neutrophils, and monocytes, leading to the attenuated release of histamine and leukotrienes as well as the release of several cytokines including IL-4, IL-5, IL-10, and granulocyte-macrophage colony-stimulating factor (Schmidt et al., 2001). Inhibition of PDE4 increases accumulation of intracellular cAMP and helps to balance anti- and pro-inflammatory effects. PDE4 inhibitors such as apremilast, roflumilast, and crisaborole have been tested in clinical trials for various inflammatory diseases. Apremilast is now approved for the treatment of adults with moderate to severe plaque psoriasis and/or psoriatic arthritis (Pincelli et al., 2018). Roflumilast has shown initial efficacy for treating asthma, COPD, and asthma-COPD overlap (Zhang et al., 2018). Based on phase III trials, crisaborole is considered an efficacious topical agent with a safety profile and limited systemic exposure. It is promising candidate for the treatment of atopic dermatitis (Woo and Kuzel, 2019).

PDE4 inhibitors, such as roflumilast, are able to suppress various inflammatory responses (Page and Spina, 2011; Urbanova et al., 2017). A clinical study with oral doses of roflumilast in patients with COPD took place in III trial phases demonstrated its anti-allergic and anti-inflammatory benefits (Page and Spina, 2011). Roflumilast is now approved for treatment of COPD (Heffler et al., 2019) and is recommended at a dose of 500 μg once daily. Many more studies have tested the effects of roflumilast on COPD than on AR (Cilli et al., 2019).

The efficacy and safety profile of roflumilast shows that it leads to more side effects than other PDE4 inhibitors administered intranasally in patients with COPD. The most frequent side effects are nausea, diarrhea, appetite loss and weight loss, abdominal pain, headache, gastrointestinal, and sleep disturbance. These side effects limit the use of roflumilast in clinical practice. Therefore, real-world studies on the clinical use of this PDE4 inhibitor are limited (Cilli et al., 2019). No effects on the cardiovascular system have been observed (Page and Spina, 2011).

The first study to evaluate the efficacy of roflumilast in the treatment of AR was conducted by Schmidt et al. (2001). In that study, roflumilast was found to be safe and well tolerated at the same dose as that used in COPD, 500 µg once a day. Headache was the most common side effect, and was followed by nausea and dizziness in some patients. Three days after the onset of treatment, increased airflow at rhinomanometry was recorded. After 4 d of treatment, subjective improvement of the obstruction was reported by the patients. Taken together, this study provided evidence that orally administered roflumilast is an effective anti-allergy therapy in AR. No further studies have been conducted on the efficacy of roflumilast in AR (Heffler et al., 2019).

## Novel PDE4 Inhibitors

The clinical development of PDE4 inhibitors has recently focused on the novel PDE4 inhibitor CHF6001. It has been designed for intranasal delivery which allows CHF6001 to reach therapeutic concentration in the lung and reduce systemic side effects. CHF6001 administered twice a day decreases the levels of various inflammatory biomarkers in the sputum and reduces systematic adverse reactions in COPD patients (Singh et al., 2019). It seems to be well tolerated and able to reduce the allergen challenge response in asthmatic patients (Singh et al., 2016). CHF6001 is characterized by anti-inflammatory effects through the ability to reduce the activation of innate immune cells. This substance can effectively inhibit the release of TNF-α and oxidative burst.

GSK256066, another PDE4 inhibitor for intranasal use, was administered for 7 d in a double blind, placebo controlled, cross-over study. It significantly reduced the response to allergen challenge and attenuated the decrease in lung function in patients with asthma. Moreover, it is associated with low systemic effects (Singh et al., 2010). A clinical trial on cilomilast was disappointing because strong side effects such as nausea, vomiting, headache, and diarrhea were observed (Parnham et al., 2019).

## Therapeutic Use of PDE4 Inhibitors in Disorders Other Than AR

PDE4 inhibitors can also be used for the treatment of Th1-mediated autoimmune diseases. They inhibit T-cell proliferation. Compared to GCs, the activity and effects of PDE4 inhibitors appear to be better in patients with a predisposition to atopic reactions (Schmidt et al., 2001). For skin diseases, apremilast, a selective PDE4 inhibitor administered orally, is approved for the treatment of adults with plaque psoriasis and psoriatic arthritis in Japan and United States (Keating, 2017).

In a prospective study, genetic analysis revealed significant differences between the *PDE4D* (rs1588265) gene in patients with no history of nasal polyposis (a type of AR) and patients with nasal polyposis, indicating the potential of using a PD4 inhibitor for the management of patients with nasal polyposis (Apuhan et al., 2013).

The effects of PDE inhibitors on ciliary beating frequency in rabbit maxillary sinus and trachea have been also investigated. The authors compared rolipram, a cAMP-specific PDE4 inhibitor with bronchodilator and anti-inflammatory activities with milrinone, a cGMP-specific PDE3 inhibitor with good bronchodilator activity but weak anti-inflammatory activity and zaprinast, a cGMP-specific PDE5 inhibitor with weak bronchodilator effects. All of them stimulated ciliary beating frequency in explants from the maxillary sinus as well as from the tracheal mucosa (Cervin and Lindgren, 1998).

## Conclusions

AR is a common health problem with a rising incidence. Inadequate treatment may cause chronic AR and contribute to lower respiratory tract diseases such as bronchial asthma. In general, the disease can be well controlled with adequate treatment with the exception of AR, which is not mediated by the Th2-inflammatory pathway and does not respond to current available treatment modalities such as corticosteroids and antihistamines. PDE4 inhibitors can suppress a variety of inflammatory cell functions that are known to contribute to AR. However, use of systemically delivered PDE4 inhibitors, such as roflumilast, are limited by systemic side effects. Topical treatment with inhaled PDE4 inhibitors may be a viable alternative to increase tolerability and determine the maximum therapeutic potential of PDE4 inhibition in AR.

## Author Contributions

VJ and HP wrote the substantial part of the manuscript. EB contributed to the epidemiology and pathophysiology of allergic rhinitis. VC contributed to the final version of the manuscript. AC conceived of the presented idea. AC and AH reviewed the article and made contributions according to their expertise.

## Funding

VEGA 1/0055/19 and APVV-17-0250.

## Conflict of Interest

The authors declare that the research was conducted in the absence of any commercial or financial relationships that could be construed as a potential conflict of interest.
